# Assessing adult attachment after out-of-hospital cardiac arrest: an exploratory analysis and construct validation of the ECR-RS

**DOI:** 10.1016/j.resplu.2025.101209

**Published:** 2025-12-24

**Authors:** Nanna Hansen, Cæcilia von Tangen Gehrt Sivertsen, Dea Siggaard Stenbæk, Mitti Blakoe, Camilla Dichman, Bo Gregers Winkel, Anders Wieghorst, Britt Borregaard, Mette Kirstine Wagner

**Affiliations:** a Department of Clinical Research, Faculty of Health Sciences, University of Southern Denmark, Odense, Denmark; b Neurobiology Research Unit, Copenhagen University Hospital, Rigshospitalet, Copenhagen, Denmark; c Department of Psychology, University of Copenhagen, Copenhagen, Denmark; d Department of Anaesthesia and Intensive Care, Bispebjerg and Frederiksberg Hospital, Copenhagen, Denmark; e Department of Cardiology, Copenhagen University Hospital, Rigshospitalet, Copenhagen, Denmark; f Department of Cardiology, Odense University Hospital, Odense, Denmark

**Keywords:** Resuscitation, Psychometric, Patient-reported outcome, Interpersonal relations, Cross-sectional study

## Abstract

**Aim:**

To explore the construct validity of the ‘Experience in Close Relationships – Relationship Structures’-questionnaire (ECR-RS) in a population of out-of-hospital cardiac arrest (OHCA) survivors. Objectives were to (i) describe item- and scale-level response patterns, and (ii) evaluate the preliminary construct validity of the ECR-RS, including its dimensional (structural), known-groups, and convergent validity.

**Methods:**

An exploratory cross-sectional validation study, with OHCA survivors completing the ECR-RS, Hospital Anxiety and Depression Scale (HADS), and the mental health component from the Short Form-12 (SF-12 MCS) three months post- arrest. Descriptive statistics and floor/ceiling analyses were performed. Dimensional validity was assessed using response distribution patterns and exploratory factor analysis (EFA), followed by reliability using Cronbach’s *ά*s. Known-group validity was tested using a priori hypotheses, Spearman’s correlations, and Mann-Whitney U tests. Convergent validity was evaluated by correlating ECR-RS total scores with HADS and SF-12 MCS.

**Results:**

Among 123 survivors (median age 59.9 years, 84 % male), ECR-RS total scores were low on both subscales and floor effects were observed at scale level (31 % for avoidant and 72 % for anxious attachment). EFA supported the expected two-factor structure, though item 4–6 showed poor loadings/cross-loadings. Internal consistency was acceptable (total scale Cronbach’s *α* = 0.88) and improved when problematic items were excluded. Known-group hypotheses were not supported. Anxious attachment correlated moderately with symptoms of anxiety and depression and was inversely correlated with mental health scores.

**Conclusion:**

The ECR-RS demonstrated partial construct validity among OHCA survivors, but item-level inconsistencies and pronounced floor effects limit its utility. Findings highlight the need for a revised instrument better suited to post-cardiac arrest relational and psychological dynamics.

## Introduction

The sudden and traumatic nature of out-of-hospital cardiac arrest (OHCA) often leads to a lasting impact for survivors including a wide range of secondary consequences that disrupt daily life activities.^1^ Survivors commonly report cognitive impairments, fatigue, emotional distress, and reduced quality of life.^1^, ^2^ These difficulties have the potential to negatively affect survivors’ overall recovery outcomes, which might extend to their close relatives and affect family dynamics.^3^ At the same time, relatives often experience long-term psychological distress, including anxiety, depression, and caregiver strain.^4^, ^5^, ^6^ Although post-arrest recovery unfolds within the survivor-relative dyad, knowledge about the relationship dynamics remains limited, including an understanding of underlying psychological patterns, such as perceived attachment in close relationships among OHCA survivors.^7^, ^8^

The concept of attachment was originally developed by John Bowlby, who suggested that emotional development and interpersonal functioning are formed in early childhood through interactions with primary relatives.^9^ Building on this framework, Mary Ainsworth and colleagues identified distinct attachment styles, showing that early relational experiences shape children's distress responses and influence emotional regulation later in life.^6^, ^9^, ^10^ Overall, attachment theory distinguishes between secure and insecure attachment styles, meaning that secure attachment leads to emotional stability, while insecure attachment is linked to relational and emotional difficulties.^6^, ^11^ Insecure attachment styles can be further divided into avoidant and anxious interpersonal behavioural patterns.^12^ Currently, it is well known that attachment impacts individuals at multiple levels, including coping styles and interpersonal reactions to distress and strain.^13^ Importantly, insecure attachment is linked to maladaptive affect regulation strategies, which increase vulnerability to psychological stress following a highly stressful or traumatic event,^13^, ^14^ such as OHCA.^14^, ^15^

Several instruments have been developed to measure adult attachment, including the Experience in Close Relationship – Relationship Structures questionnaire (ECR-RS).^14^ The ECR-RS has been used across various populations, but due to cultural and situational influences, it is unknown whether this tool accurately captures attachment difficulties in OHCA survivors. This raises important questions about the construct validity of ECR-RS in this specific context, where existing assumptions about attachment patterns may not fully apply.

Thus, we aimed to explore the construct of adult attachment in survivors of OHCA using the ECR-RS with the specific objectives to (i) describe item- and scale-level response patterns, and (ii) evaluate the preliminary construct validity of the ECR-RS, including its dimensional (structural), known-groups, and convergent validity.

## Method

### Study design, setting and participants

This exploratory cross-sectional validation study is based on a secondary analysis of cohort data from the REVIVAL study. The design, methodology, and eligibility criteria are detailed in the published protocol.^16^ In brief, data were collected from first-time OHCA survivors at three specialised heart centres in Denmark between January 2018 and February 2022. Participants were recruited during hospitalisation and provided informed consent.

### Data collection and outcome measures

Sociodemographic and clinical data were obtained from OHCA survivors during hospitalisation and at three-month follow-up, where both survivors and relatives received a survey consisting of several patient-reported outcome measures (PROMs). The survey was sent via a token specific email and completed online. In the current substudy, three PROMs from the survivor survey were included:

The **ECR-RS** assesses adult attachment through the two subscales, avoidant and anxious attachment. The instrument covers 9 items (items 1–6 form the avoidant subscale, and items 7–9 the anxious subscale). Items are rated on a 7-point Likert scale ranging from 1 (strongly disagree) to 7 (strongly agree) with four items being reverse scored (Items 1–4) when calculating the two subscales. The scores for each subscale represent mean values of the relevant items. The ECR-RS has no validated cut-off scores indicating insecure relationships and can be used across various relationship types.^17^

The Hospital Anxiety and Depression Scale (**HADS**) is an internationally validated questionnaire containing 14 items, with 7 items assessing symptoms of anxiety (HADS-A) and 7 for depression (HADS-D). HADS is a commonly used instrument to screen for mood disorders in cardiac populations, with higher scores reflecting increased anxiety and depression symptoms.^18^, ^19^, ^20^ Importantly, the scale has also demonstrated sound psychometric properties specifically among cardiac arrest survivors, further supporting its suitability for use in the present study.^21^

The Short Form Health Survey-12 Mental Component Score (**SF-12 MCS**) measures overall mental health status. SF-12 MCS is scored using a weighted algorithm, where responses are transformed to a 0–100 scale with higher scores indicating better mental health.^22^, ^23^

### Psychometric properties

This study evaluated the construct validity of the ECR-RS by examining its dimensional (structural), known-groups and convergent validity. Item- and scale level descriptive analyses were conducted to assess response distribution. Further, visualisations of results included boxplots, histograms and a heatmap to explore score distribution and potential floor or ceiling effects.^24^, ^25^ Floor and ceiling effects were considered present if more than 15 % of respondents achieved the lowest or highest possible score, respectively, in accordance with established psychometric criteria.^25^

### Construct validity assessment

Construct validity was assessed through two approaches: quadrant classification and exploratory factor analysis (EFA). First, participants’ mean scores on the two ECR-RS subscales were used to classify attachment style according to Bartholomew’s four-style model (the quadrant classification), using a median split at four on each axis.^26^ Secondly, an EFA was conducted to assess the underlying factor structure of the ECR-RS items and to identify clusters of intercorrelated variables. An exploratory rather than confirmatory approach was chosen due to the lack of prior validation of the ECR-RS in OHCA survivors, pronounced floor effects, and the exploratory nature of assessing dimensionality in this context.

### Known-groups validity

Known-groups validity was assessed based on a priori hypotheses from prior empirical research, in line with COSMIN recommendations for evaluating construct validity.^27^ We hypothesised that OHCA survivors living with a partner would report lower attachment-scores, as romantic relationships are linked to more secure attachment patterns.^28^ We also expected higher insecurity among participants with fewer years of education or a history of psychiatric diagnosis,^9^, ^29^ as well as gender differences characterised by higher avoidance in men and higher anxiety in women.^30^, ^31^

### Convergent validity

We compared the ECR-RS with established outcome measurements, including HADS-A, HADS-D, and SF-12 MCS. We hypothesised that anxious attachment would be positively correlated with HADS-A and negatively correlated with SF-12 MCS.^32^, ^33^

### Internal consistency

Internal consistency was assessed using Cronbach’s *α*. A previous ECR-RS study in non-cardiac populations demonstrated high internal consistency with Cronbach’s *α* values of 0.94 for avoidant attachment and 0.91 for anxious attachment, respectively.^34^

### Statistical methods

Principal axis factoring with oblique (promax) rotation was used to conduct the EFA. The oblique rotation was applied to allow the correlations between factors, as the attachment dimensions are theoretically expected to be correlated (interrelated rather than independent), consistent with attachment theory. Factors were retained based on eigenvalues >1.^35^, ^36^ When analysing the factor loadings, a loading >0.70 was considered strong, loadings >0.40 were deemed acceptable, while loadings <0.30 indicated a poor factor fit.^37^ To evaluate factorability, the Kaiser-Meyer-Olkin (KMO) test and Bartlett’s Test of Sphericity were assessed. A KMO value ≥0.6 and a significant Bartlett́s Test of Sphericity with *p* < 0.05 indicate that the data are suitable for factor analysis.^38^

To examine known-groups validity, ECR-RS scores (median and interquartile range, [IQR]) were compared across subgroups using the Mann-Whitney *U* test. Spearman’s rank correlation was applied to examine correlations between ECR-RS and the selected sociodemographic and clinical characteristics, including self-reported prior psychiatric diagnoses.

Spearman’s rank correlation was applied to test convergent validity, due to non-normal distribution of PROMs data.

In addition to the above psychometric analyses, baseline sociodemographics, and clinical variables are reported as numbers and proportions (%) for categorical variables, whereas continuous variables are reported as mean, standard deviations and range or median and IQR (25th–75th percentile), as appropriate.

Data were analysed using STATA 18.0 (StataCorp, 2023), and a *p*-value < 0.05 was considered statistically significant.^39^ The study followed STROBE guidelines.^39^

### Ethics and dissemination

The REVIVAL study was approved by the Regional Research Ethic Committee (H-18046155). Handling of data was approved by the Data Protection Agency (journal no: RH-2017-325).

## Results

### Participants, demographics, and clinical characteristics

During the study period, 287 OHCA survivors were screened during hospitalisation, and 205 participated in the three months follow-up, and thus were eligible for inclusion in the current study. Of the eligible population, 123 survivors responded to the ECR-RS at three months (response rate 60 %) (Supplementary Fig. S1). The median age of the responding survivors was 59.9 years (IQR 51–67), 84 % were male, and 94 % were living with a partner (Table 1). In total, 70 % had ≥12 years of education, and 20 % reported a history of psychiatric diagnosis prior to the OHCA.

**Table 1:** Baseline sociodemographics and clinical characteristics of respondents.

**Survivor characteristics**	***n* = 123**
**Age, years**	
Mean (SD), range	59.3 (11.6), 25–86
Median (IQR)	59.9 (51–67)
Male, sex, *n* (%)	103 (84)
Living with partner or another close relative, *n* (%)	114 (93)
Education ≥12 years, *n* (%)	85 (70)
**OHCA-place, *n* (%)**	
Home	34 (28)
Public space	54 (43)
Other, unknown	35 (29)
**Time to ROSC, min**	
Median (IQR)	12 (9–20)
Psychiatric diagnoses prior to OHCA, *n* (%), yes	25 (20)
**Length of coma duration, h**	
Median (IQR)	28 (0–46)
Stayed at ICU, *n* (%), yes	99 (68)
**Length of ICU stay, h**	
Median (IQR)	50 (1–89)
ICD implanted, *n* (%), yes	79 (64)
**Length of hospital stay, days**	
Median (IQR)	12 (8–16)

**OHCA:** out-of-hospital cardiac arrest, **ICU**: intensive care unit, **ROSC**: return of spontaneous circulation, **ICD**: implantable cardioverter defibrillator, **IQR**: interquartile range, **SD**: standard deviation. Psychiatric diagnoses prior to OHCA were self-reported data.

### Dimensional (structural) validity and reliability

Descriptive data of the ECR-RS are presented in Supplementary Table S1. The mean score for avoidant attachment was 2.03 (*SD =* 1.12) and 1.50 (*SD =* 1.15) for anxious attachment. Floor effects were evident, with 31 % of respondents exhibiting a mean score of 1 on the avoidant attachment subscale, and 72 % exhibiting a mean score of 1 on the anxious attachment subscale. Supplementary Fig. S2 depicts boxplots of the two dimensions, with scores being low related to both avoidant attachment: median 2.06 (IQR 1.0–2.7), and anxious attachment: median 1.0 (IQR 1.0–1.3).

**Fig. 1:**
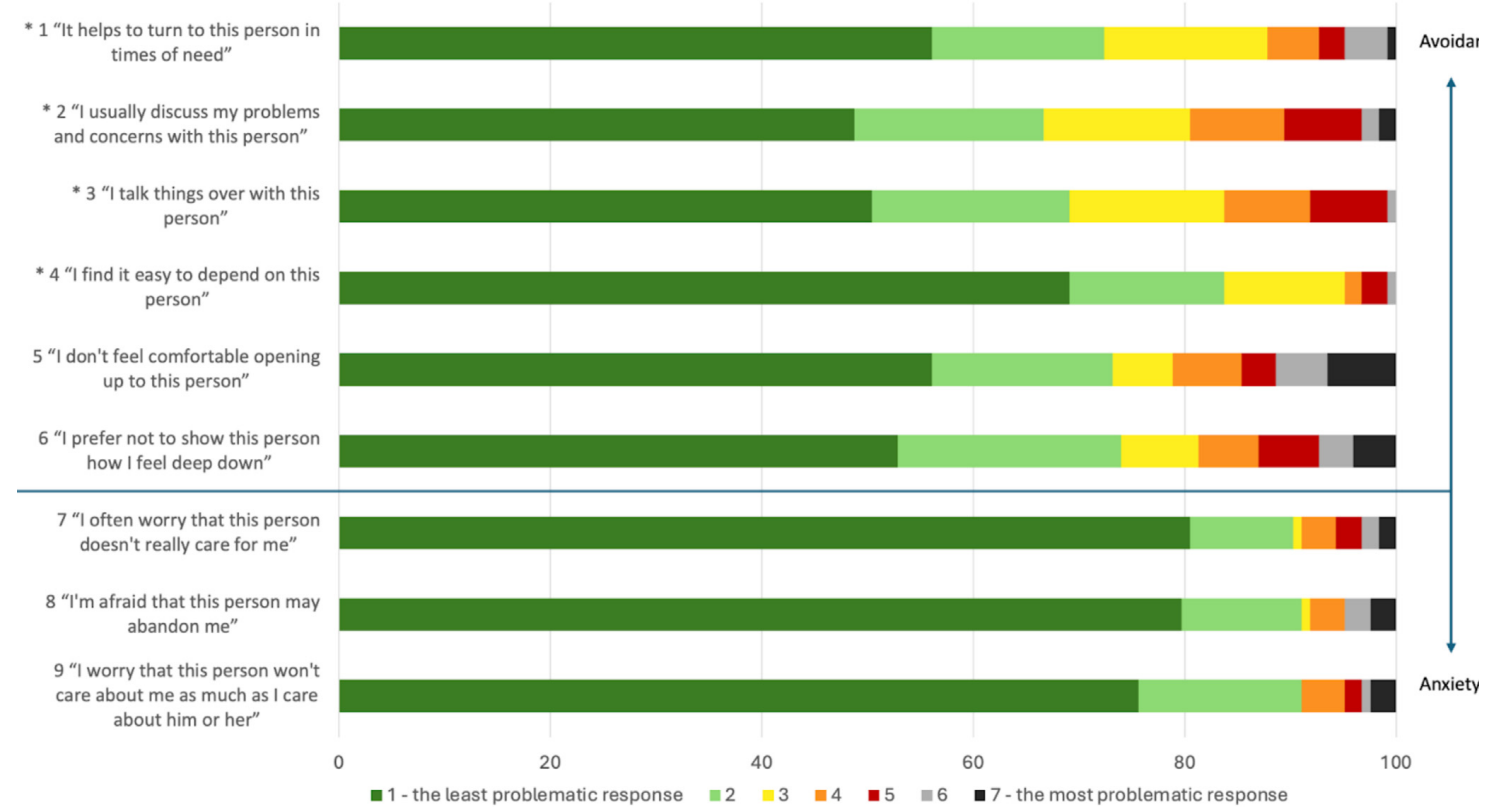
**Distribution of answers in the ECR-RS in percent**. Each item is scored on a scale of 1–7. Item 1–4 (highlighted with *) are reversed scores, when summing the two subscales. 1 marks the least problematic responses, while 7 marks the most problematic response.

The distribution of responses across ECR-RS items, expressed in percentages, are presented in Fig. 1. Overall, responses were predominantly concentrated at the lower end of the 1–7 Likert scale, showing floor effects across most items. To further assess the distribution pattern of answers, Supplementary Fig. S3 displays a heatmap highlighting this pattern, confirming that the majority of survivors reported low levels of both avoidance and anxiety, with most responses falling between scores 1 and 2. Items 7–9 (anxious attachment), showed less frequent use of higher response categories compared to avoidance items, although some variations were observed across items. Items 5 and 6 had the highest number of elevated (worse) responses, with 7 % of the survivors selecting the highest score (score 7 = worst) on item 6, in contrast to the predominantly low responses elsewhere. Items 7 and 8 showed a strong skew toward the lowest response option (80 %), underscoring the overall floor effect observed when using ECR-RS in OHCA survivors (Fig. 1).

**Fig. 2:**
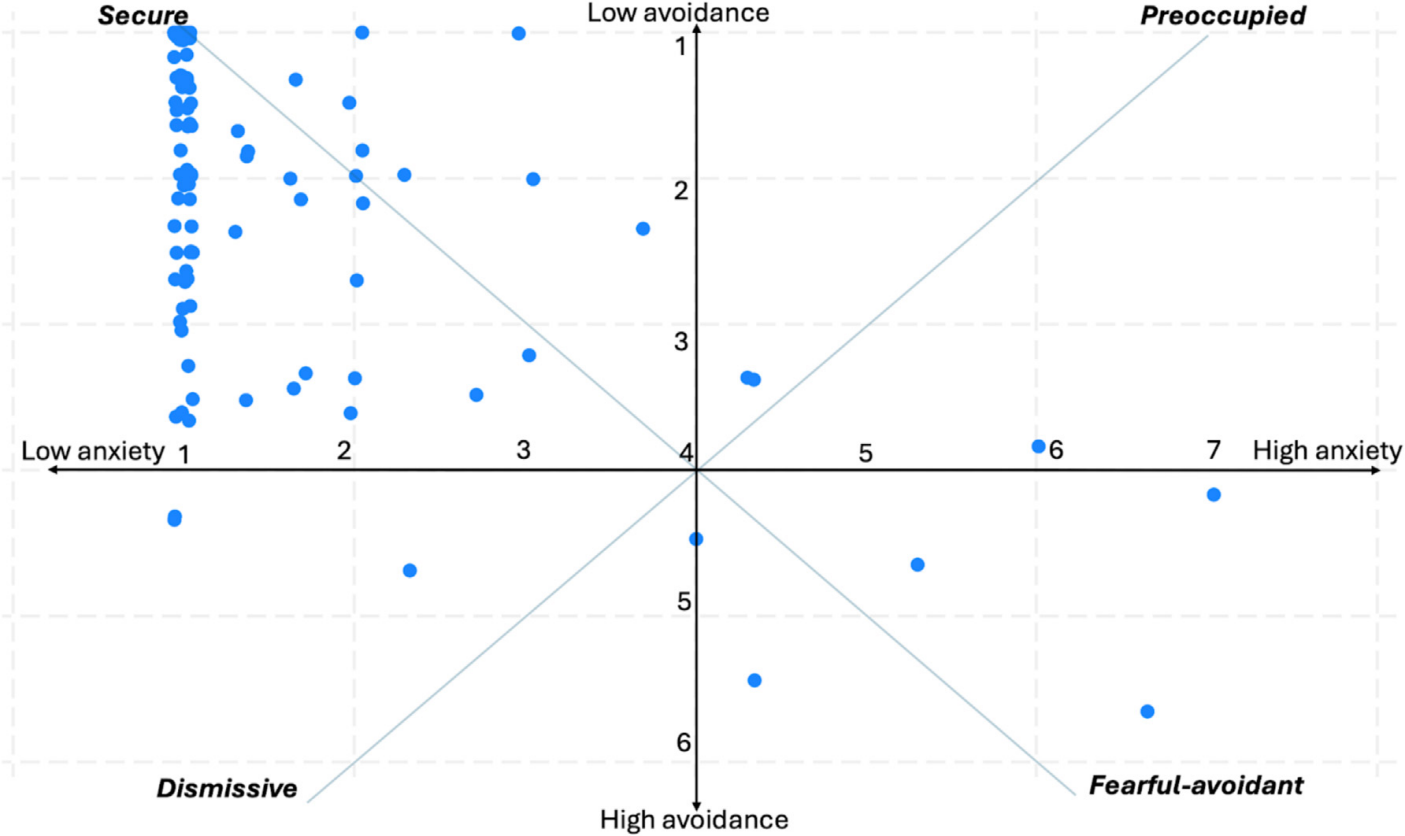
**Scatterplot with Bartholomew's four adult attachment style quadrants**. Distribution of experience in close relationship-relationship structures responders across Bartholomew's four quadrants model for adult attachment. A cut-off at 4 was applied on both axes according to Batholomew’s theory.

Fig. 2 presents the distribution of answers across Bartholomew’s four quadrants, based on mean ECR-RS avoidance and anxiety scores. The majority of participants clustered in the secure quadrant. Avoidance scores demonstrated greater variability across the sample, while anxiety scores were strongly skewed toward the minimum value (score 1 = best).

Based on the EFA (Table 2), including factors with an eigenvalue >1 and the scree plot (Supplementary Fig. S4), a two-factor solution consistent with the theoretical model was identified. Factor 1 showed a strong eigenvalue of 4.61, while Factor 2 had a weaker but acceptable eigenvalue of 1.18. The KMO value was 0.50, indicating marginally suitability for factor analysis, and Bartlett’s Test of Sphericity was significant (*p* ≤ 0.001), supporting that the correlation matrix was appropriate for factor analysis.

**Table 2:** Exploratory factor loading on each question of the ECR-RS questionnaire.

**Item**		**Factor 1**	**Factor 2**	**Communalities**
1	*“It helps to turn to this person in times of need”*	**0.90**	−0.01	0.83
2	*“I usually discuss my problems and concerns with this person”*	**0.98**	−0.10	0.86
3	*“I talk things over with this person”*	**0.94**	0.02	0.90
4	*“I find it easy to depend on this person”*	0.42	**0.44**	0.57
5	*“I don’t feel comfortable opening up to this person”*	−0.05	**0.49**	0.26
6	*“I prefer not to show this person how I feel deep down”*	**0.38**	0.10	0.24
7	*“I often worry that this person doesn’t really care for me”*	−0.05	**0.87**	0.71
8	*“I’m afraid that this person may abandon me”*	−0.04	**0.89**	0.73
9	*“I worry that this person won’t care about me as much as I care about him or her”*	0.07	0.**83**	0.72
	Cronbach Alpha Factor 1	0.81		
	Cronbach Alpha Factor 2		0.83	
	Cronbach Alpha total	0.88		

	Bartlett’s test of Sphericity	*p*-value < 0.001		
	Kaiser-Meyer-Olkin test	0.5		

Exploratory Factor analysis using principal axis extraction based on eigenvalues >1. Oblique (promax) rotation, cut-off >0.70 was considered strong, >0.40 acceptable and <0.30 indicating low factor fit. Primary factor loadings are shown in bold.

Communalities were examined to assess the proportion of variance explained by the factors, and items with communalities below 0.30 were considered poorly represented.

Factor 1 included strong loadings from item 1 (0.90), 2 (0.98), and 3 (0.94), aligning with the expected structure. Factor 2 was defined by item 7 (0.87), 8 (0.89) and 9 (0.83), also reflecting the expected factor grouping. However, some items showed unexpected loading patterns. Item 4 cross-loaded on both factors (0.42 and 0.44) and item 5 loaded moderately to factor 2 (0.49), although expected to load on factor 1. Item 6 loaded weakly to factor 1 (0.38). Item communalities indicated strong representation for items loading clearly on each factor, whereas items 4–6 showed lower communalities, consistent with their weaker and cross-loading patterns.

The two-factor structure of the ECR-RS was supported with Cronbach’s *α* values of 0.81 for factor 1, 0.83 for factor 2, and a total scale alpha of 0.88. Together, the two retained factors explained 60 % of the total variance.

Supplementary Table S2 depicts Cronbach’s *α* for the sub-scales and all individual items, including the item rest correlation of items 5 and 6 of 0.43 and 0.47 (limited alignment with all subscales). When items 5 and 6 were excluded from the overall calculation for avoidance subscale, the total alpha score increased to 0.92, indicating improvement of the internal consistency.

### Known-groups validity

No statistically significant correlations or group differences were observed between attachment scores and sociodemographic or clinical characteristics (Table 3).

**Table 3:** Known-groups validity – sociodemographic, clinical and psychosocial characteristics.

**Variable**	**ECR-RS total score, median (IQR)**		**Spearman's correlation, *p*-value**		**Mann-Whitney *U* test, *p*-value**	
	**Anxiety**	**Avoidance**	**Anxiety**	**Avoidance**	**Anxiety**	**Avoidance**
Age			0.04, *p* = 0.673	−0.05, *p* = 0.582		
Sex					0.361	0.309
Male	1 (1–1.33)	1.83 (1–2.67)				
Female	1 (1–2)	1.5 (1–2.67)				
Cohabiting with close relatives					0.565	0.285
Yes	1 (1–1.33)	1.75 (1–2.5)				
No	1 (1–2)	2.33 (1.3–3.5)				
Level of education ≥12 years					0.081	0.205
Yes	1 (1–1)	1.67 (1–2.5)				
No	1 (1–2)	2 (1.33–3)				
Previous psychiatric diagnosis OHCA					0.900	0.628
Yes	1.33 (1–1.33)	1.67 (1–2.5)				
No	1 (1–1.33)	1.83 (1–2.67)				
Previous psychiatric diagnosis relatives					0.575	0.843
Yes	1 (1–1)	1.83 (1–2.6)				
No	1 (1–1)	1.33 (1–2.17)				

**ECR-RS:** Experience in Close Relationships – Relationships Structures. The non-parametric Spearman's rank correlation coefficients were classified as weak (less than 0.30), moderate (0.30–0.50), moderately strong (0.50–0.80) and strong (0.80–1). Group differences were tested through the non-parametric Mann-Whitney *U* test. *p* < 0.05 was considered statistically significant.

### Convergent validity

Anxious attachment was weakly to moderately correlated with both HADS-A (anxiety) and HADS-D (depression) scores (Spearman’s rho 0.31 and 0.32, respectively *p* = 0.005). The ECR-RS avoidant and anxious attachment subscales were both negatively correlated with SF-12 MCS (Spearman’s rho of −0.24, *p* = 0.007 and −0.28, *p* = 0.002, respectively), indicating that higher levels of attachment insecurities were associated with poorer mental health (Table 4).

**Table 4:** Spearman's correlation between ECR-RS domains and psychological domains in OHCA survivors.

**Psychological domains**	**ECR-RS domains**	
	**Avoidant attachment style**	**Anxious attachment style**
**HADS**		
HADS-A	0.27**	0.31**
HADS-D	0.20*	0.32**

**SF-12**		
SF-12, mental health	−0.24**	−0.28**

**ECR-RS**: Experience in Close Relationships – Relationships Structures, **HADS**: Hospital Anxiety and Depression Scale, **HADS-A**: Anxiety, **HADS-D**: Depression, **SF-12**: Short-Form-Health-Survey, **MCS**: Mental Health Component. The non-parametric Spearman's rank correlation coefficients were classified as weak (less than 0.30), moderate (0.30–0.50), moderately strong (0.50–0.80) and strong (0.80–1).

** *p* < 0.01.

* *p* < 0.05.

## Discussion

This study examined the construct validity of the ECR-RS among adult OHCA survivors. Scores on both attachment dimensions were generally low, suggesting floor effects and limited variability. EFA revealed the expected two-factor structure, though items 4–6 showed cross-loadings and weak alignment, highlighting areas for refinement. Known-group validity was not supported, whereas, in contrast, weak correlations between attachment dimensions and symptoms of anxiety, depression and reduced mental health supported the convergent validity of the scale. The findings raise questions about the sensitivity of the ECR-RS in this population and point to an overlap between attachment constructs and general psychological distress, rather than a clear distinction between these constructs.

The EFA showed that items 4–6 could benefit from rewording or closer theoretical reconsideration to improve alignment with the intended construct. Nevertheless, the content of the items that successfully loaded on each factor suggests a conceptual alignment with the established constructs of attachment dimensions. Items 1–3 capture avoidance, focusing on discomfort with closeness and dependence on others. These features are core characteristics to avoidant attachment, the theorised domain for factor 1. Items 7–9 loaded on factor 2, reflecting fear of rejection and desire for stronger emotional closeness. These items correspond to anxious attachment and align with the theoretical framework for the ECR-RS construct.^40^

In the current study, items 4–6 did not load as expected on factor 1, which may indicate potential psychometric issues or contextual complexity. Interestingly, two of these items also showed the most severe response patterns, which indicates they may not be contextually irrelevant, but instead that these items capture a different underlying construct, not reflected in the identified dimensions. Three items are expressed as^4^: ‘I find it easy to depend on this person',^5^ ‘I don't feel comfortable opening up to this person’, and^6^ ‘I prefer not to show this person how I feel deep down’. Thus, the items are conceptually linked to trust, emotional openness, and communication. They may reflect interpersonal expressions of insecure attachment, such as discomfort with emotional closeness, or a distinct relational domain not captured as a dimension itself by the traditional avoidance-anxiety model,^40^ potentially reflecting disruptions in communication patterns following OHCA. The findings align with insights from a qualitative study, describing how OHCA survivors often experience a persistent sense of vulnerability.^41^ As a result, survivors may choose not to burden their loved ones with emotional and physical struggles, indicating relational challenges that arise from the cardiac arrest itself, rather than pre-existing attachment patterns.^41^ Moreover, evidence from dyadic research demonstrates that survivors’ and partners’ psychological characteristics mutually influence one another, including how Type D personality and perceived control affect both parties’ health-related quality of life.^42^ Thus, these responses may reflect both attachment-related tendencies and situational factors related to the cardiac arrest. Further qualitative research may help clarify how survivors navigate vulnerability, guilt and the perceived need to protect their loved ones from emotional strain.

Previous research on the ECR-RS has generally supported a two-factor structure, although lower item loadings and minor cross-loadings have been reported in certain non-cardiac populations.^17^, ^43^ In addition, items 4–6 have previously been shown to demonstrate weaker loadings, suggesting that these items may reflect relationship dynamics not fully captured by the original model of the ECR-RS.^44^, ^45^ The low variation in attachment scores observed in this study demonstrate floor effects. While this could potentially point to genuinely secure attachment, it could also be a result of social disability bias or cognitive impairments following OHCA, which may affect survivors insights and ability to self-report on emotional patterns.^2^, ^46^, ^47^ Our findings highlight the need for an instrument that assesses changes in relational self-perception and communication patterns that emerge after OHCA.

Construct validity was investigated through known-groups validity, where the hypothesised group differences were not supported. This may suggest insufficient sample size or limited precision in how subgroups were defined. For example, categories such as cohabitants status and educational level could be refined to improve sensitivity. The relative homogeneity of the sample may also be a contributing factor, as most participants were male (84 %) and of similar age (median, 59.9, IQR, 51–67 years), resulting in limited variation. This limits the external validity in terms of generalisation. Notably, although non-significant, survivors *without* a psychiatric history tended to report higher attachment insecurity, contrary to expectations.^2^, ^28^

The convergent validity analysis provided partial support for the construct validity of ECR-RS. Anxious attachment was moderately correlated with anxiety, as measured by HADS-A, aligning with theoretical expectations.^48^ However, it is important to note that elevated scores of anxiety measured using HADS-A may reflect a broader range of psychological stressors such as health-related worry or post-OHCA vulnerability rather that attachment-related distress.^49^ Anxious attachment was also moderately correlated with depression (HADS-D), and both avoidant and anxious attachment were negatively correlated with mental health measured by SF-12. These findings indicate that ECR-RS may, in this context, reflect a more general emotional vulnerability.^50^, ^51^

The above findings suggest that the questionnaire captures relational issues not originally theorised in the ECR-RS and further indicates that the relational challenges in OHCA survivors differ from the construct. To better understand these issues, cognitive interviews based on ECR-RS responses could help clarify how relationships are affected by a traumatic event like OHCA.^52^

### Strength and limitations

This study has several methodological strengths and limitations. Its focus on the relational impact on OHCA survivors and relatives addresses an underexplored yet clinically relevant area. By evaluating the applicability of a widely used and validated questionnaire to OHCA survivors, this study contributes to new insights into both clinical practice and research. First, selection bias is a concern, as non-responders had more comorbidities and longer Intensive Care Unit stay,^2^ possibly underrepresenting those with insecure attachment. The sample, with 84 % male participants, reflects the higher cardiac arrest incidence among men.^53^, ^54^ The small sample size, the slightly younger group and the overrepresentation of survivors in romantic relationships (94 %) compared to similar Danish populations from the DANCAS study (≈81 %)^55^ may limit generalisability and potentially explain the very high level of secure attachment.^28^, ^55^ On the anxious scale, outliers were present in 17 % (*n* = 23). Women were overrepresented in the subgroup (48 % vs. 16 % in the total sample); aligning with prior findings that women report higher anxiety.^30^, ^56^, ^57^ This suggests that gender differences in attachment are more pronounced at extreme levels.^30^ Although reverse-scored items can be problematic for some respondents, particularly among individuals with cognitive difficulties, a known secondary consequence of OHCA, our findings do not indicate such problems. As shown in Fig. 1, the reverse-scored items displayed response patterns comparable to the non–reverse-scored items, suggesting that participants generally understood the items. A limitation of this study is that we relied on classical test theory–based methods and did not apply modern psychometric approaches such as Rasch analysis or item response theory (IRT). These methods could have provided additional insights into item functioning, scale sensitivity across different levels of attachment insecurity, and measurement precision, particularly in the presence of pronounced floor effects. Future studies should apply such approaches to further evaluate item performance and the suitability of the ECR-RS in OHCA survivors.

## Conclusions

The ECR-RS demonstrated partial construct validity among OHCA survivors, but item-level and population-specific inconsistencies and pronounced floor effects limit its applicability in this population. The findings highlight the need for a revised instrument that more precisely captures the relational and psychological dynamics specific to the post-cardiac arrest context.

## Formatting of funding sources

The REVIVAL cohort was funded by The Research Fund of Rigshospitalet – Copenhagen University Hospital (grant no. E-22281-05), the Research Fund between Copenhagen University Hospital, Rigshospitalet and Odense University Hospital (grant no. R38-2015), and The Danish Health Foundation (grant no. 18-B-0235). The current substudy did not receive any specific grant from funding agencies in the public, commercial, or not-for-profit sectors.

## CRediT authorship contribution statement

**Nanna Hansen:** Writing – original draft, Formal analysis, Conceptualization. **Cæcilia von Tangen Gehrt Sivertsen:** Writing – original draft, Formal analysis, Conceptualization. **Dea Siggaard Stenbæk:** Writing – review & editing, Methodology, Data curation. **Mitti Blakoe:** Writing – review & editing. **Camilla Dichman:** Writing – review & editing. **Bo Gregers Winkel:** Writing – review & editing. **Anders Wieghorst:** Writing – review & editing. **Britt Borregaard:** Writing – original draft, Supervision, Methodology, Investigation, Conceptualization. **Mette Kirstine Wagner:** Writing – original draft, Supervision, Methodology, Investigation, Funding acquisition, Data curation.

## Declaration of competing interest

The authors report no conflicts of interest. The authors alone are responsible for the content and writing of the paper. Due to the General Data Protection Regulation, the data that support the findings of this study are not readily available.
