# Supplementary material for: Assessing adult attachment after out-of-hospital cardiac arrest: an exploratory analysis and construct validation of the ECR-RS
Source: Resusc Plus. 2025 Dec 24;27:101209. doi: 10.1016/j.resplu.2025.101209 (PMC12811464; doi:10.1016/j.resplu.2025.101209)
Supplement: Supplementary Data 1 [file mmc1.docx]

**Supplementary files**

**Figure S1:** Flowchart over participants

**Table S1:** ECR-R scale level descriptive statistics

**Figure S2:** Boxplots of avoidance and anxiety attachment

**Figure S3:** Heatmap of ECR-R item responses (Visualises % response per Likert scale option).

**Table S2:** Cronbach’s α of ECR-RS domains

**Figure S4:** Scree plot of eigenvalues

**Table S3:** Outliers sociodemographics and clinical characteristics


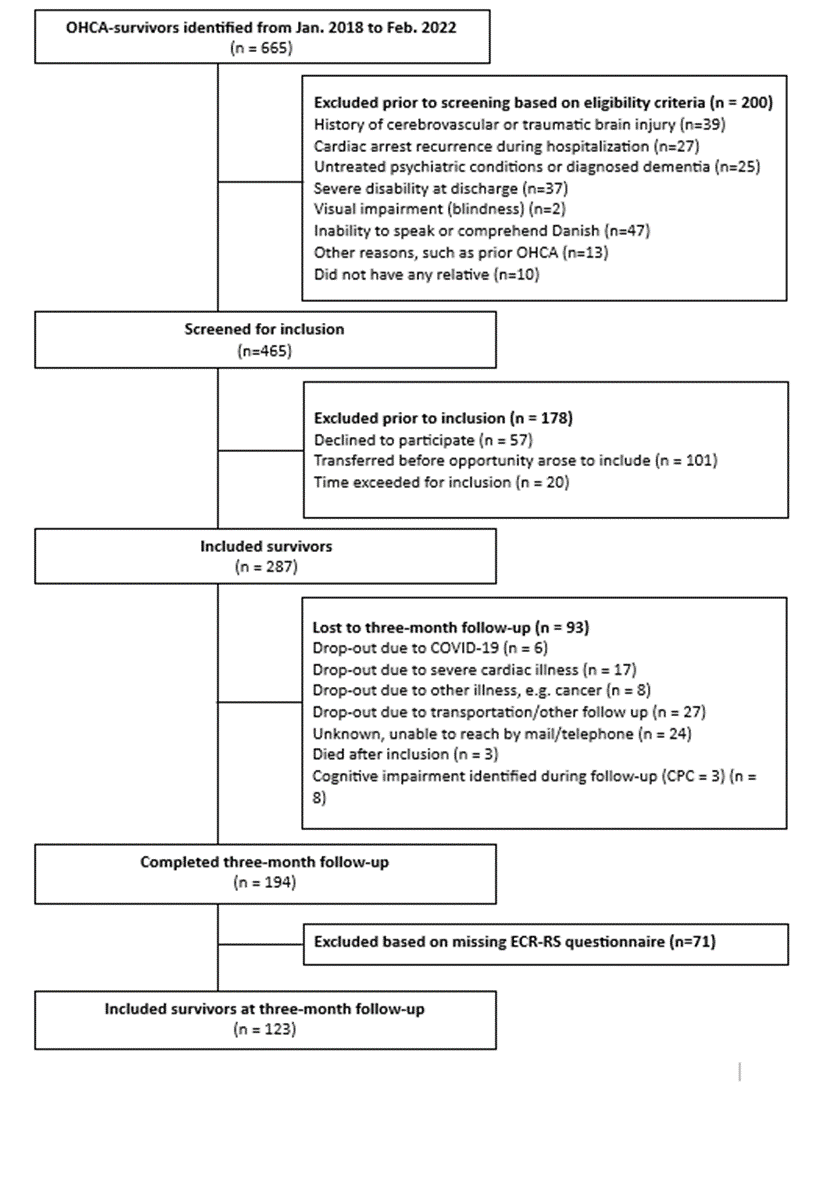


**Figure S1.** Flowchart over participants

**Table S1.** ECR-R scale level descriptive statistics

|  |  |  |  |  | **n** = 123 |
| --- | --- | --- | --- | --- | --- |
| **Variables** | **Mean** | **Responders with mean=1** | **Std. dev.** | **Median** | **IQR** |
| ECR-RS, avoidance | 2.03 | 38 (31%) | 1.12 | 1.83 | 1-2.7 |
| ECR-RS, anxiety | 1.50 | 89 (72%) | 1.15 | 1 | 1-1.3 |

**SD:** Standard deviation, **IQR**: Interquartile range, **ECR**-**RS**: Experience in Close Relationship - Relationship Structures.

**Figure S2.** Boxplots of avoidance and anxiety attachment

**
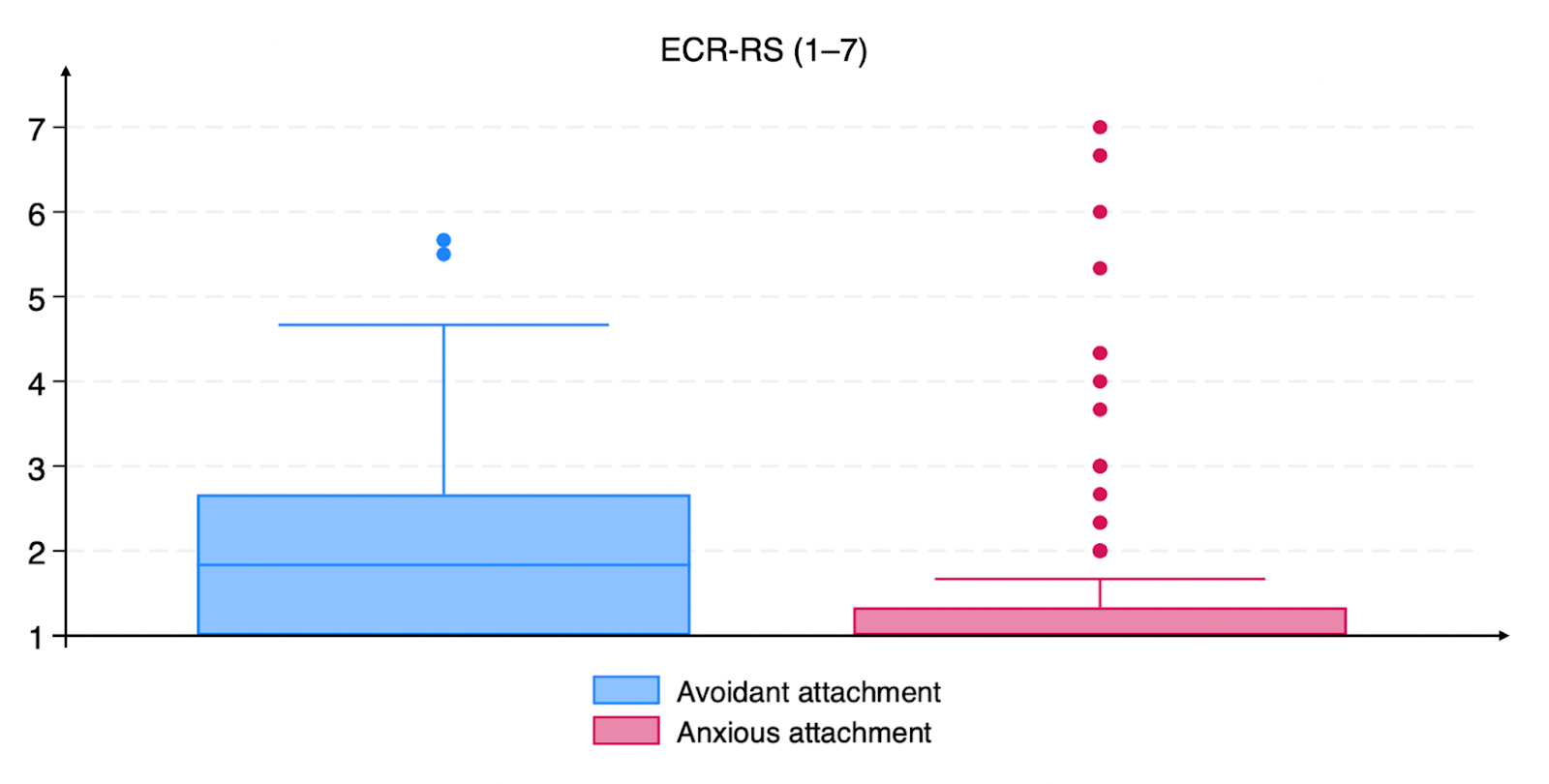
**

**ECR-RS:** Experience in Close Relationships - Relationships Structures. Boxplot shows the median (line), IQR (box), minimum and maximum values within 1.5 x IQR (whiskers). Outliers are shown as individual points.

**Figure S3.** Heatmap of ECR-R item responses (Visualises % response per Likert scale option).


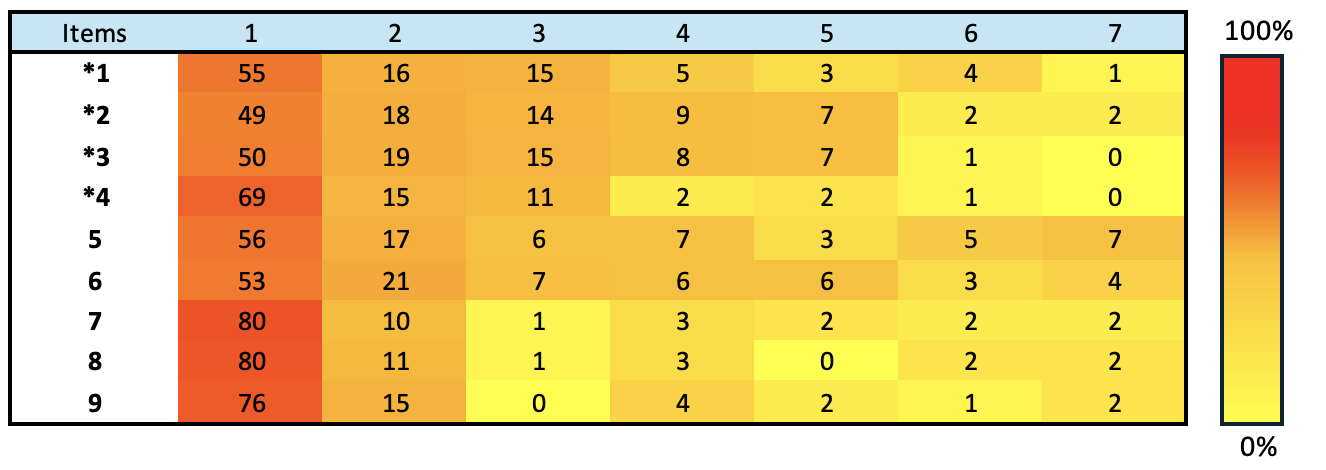


**ECR-RS**: Experiences in Close Relationships - Relational Structures.  Each item is scored on a scale of 1-7. Item 1-4 (highlighted with *) are reversed scores, when summing the two subscales. 1 marks the least problematic responses, while 7 marks the most problematic responses.

**Table S2.** Cronbach’s α of ECR-RS domains

| **ECR-RS domains** | **Item-rest correlation** | **Cronbach's** α |  |
| --- | --- | --- | --- |
| **1** | 0.731 | 0.853 |  |
| **2** | 0.688 | 0.857 |  |
| **3** | 0.772 | 0.850 |  |
| **4** | 0.704 | 0.861 |  |
| **5** | 0.430 | 0.888 |  |
| **6** | 0.466 | 0.880 |  |
| **7** | 0.667 | 0.860 |  |
| **8** | 0.642 | 0.862 |  |
| **9** | 0.684 | 0.859 |  |
| **1-6,** total score (avoidance) |  | 0.830 |  |
| **7-9,** total score (anxiety) |  | 0.892 |  |

**ECR-RS:** Experiences in Close Relationships - Relational Structures. Internal consistency (Cronbach's α) for the questionnaire ECR-RS. Values >0.70 were acceptable.

**Figure S4.** Scree plot of eigenvalues


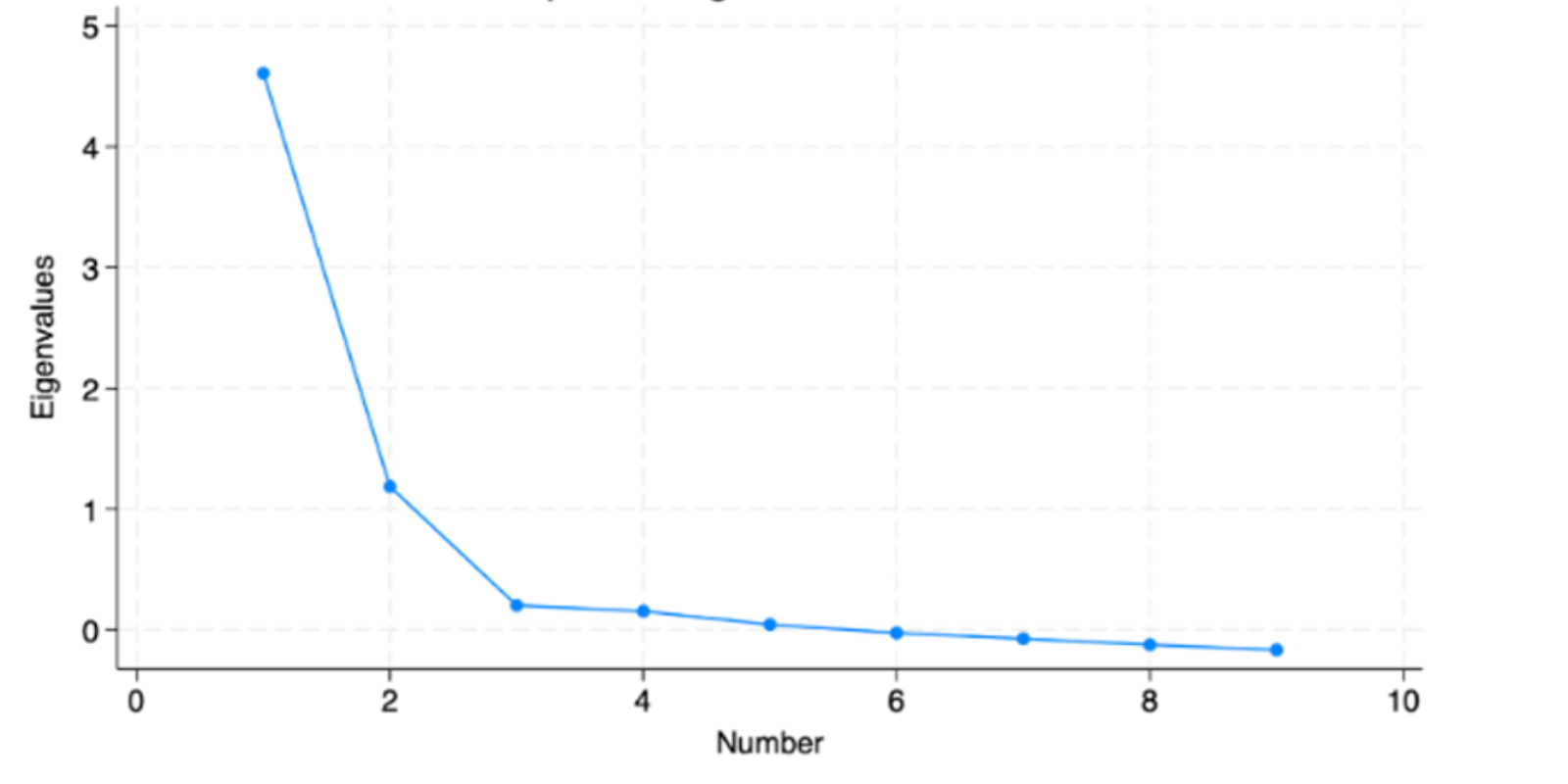


Factors with eigenvalues greater than 1 were applied for inclusion.

**Table S3.** Outliers sociodemographics and clinical characteristics

| **n = 23** | |
| --- | --- |
| **Age (mean), years** | 61.7 |
| SD, range | 12.8 (41-86) |
| **Sex, male, n %** | 12 (52) |
| **Previous psychiatric diagnosis, survivor, n (%)** |  |
| Yes | 4 (17) |
| No | 19 (83) |
| **Previous psychiatric diagnosis, relatives, n (%)** |  |
| Yes | 3 (13) |
| No | 20 (87) |
| **Cohabiting with partner or another close relative, n (%),** yes | 20 (87) |
| **HADS, survivors,** mean (SD) |  |
| HADS-A | 6.5 (5.9) |
| HADS-D | 5.1 (5) |
| **SF-12, mental health,** mean (SD) | 42 (15.1) |
| *Remaining study population (-outliers)* | **n = 100** |
| **HADS, survivors,** mean (SD) |  |
| HADS-A | 3.2 (3.2) |
| HADS-D | 2.7 (3.3) |
| **SF-12, mental health,** mean (SD) | 49.7 (11) |

**OHCA:** out-of-hospital cardiac arrest, **ICU**: Intensive Care Unit, **ROSC**: return of spontaneous circulation, **ICD**: Implantable cardioverter defibrillator. Data are presented as mean ± standard deviation (SD), median and interquartile range (IQR) and number (percent
